# An enhanced nonparametric EWMA sign control chart using sequential mechanism

**DOI:** 10.1371/journal.pone.0225330

**Published:** 2019-11-21

**Authors:** Muhammad Riaz, Muhammad Abid, Hafiz Zafar Nazir, Saddam Akber Abbasi

**Affiliations:** 1 Department of Mathematics and Statistics, King Fahad University of Petroleum and Minerals, Dhahran, Saudi Arabia; 2 Department of Statistics Government College University Faisalabad, Pakistan; 3 Department of Statistics University of Sargodha, Sargodha, Pakistan; 4 Department of Mathematics, Statistics and Physics, Qatar University, Doha, Qatar; Shandong University of Science and Technology, CHINA

## Abstract

Control charts play a significant role to monitor the performance of a process. Nonparametric control charts are helpful when the probability model of the process output is not known. In such cases, the sampling mechanism becomes very important for picking a suitable sample for process monitoring. This study proposes a nonparametric arcsine exponentially weighted moving average sign chart by using an efficient scheme, namely, sequential sampling scheme. The proposal intends to enhance the detection ability of the arcsine exponentially weighted moving average sign chart, particularly for the detection of small shifts. The performance of the proposal is assessed, and compared with its counterparts, by using some popular run length properties including average, median and standard deviation run lengths. The proposed chart shows efficient shift detection ability as compared to the other charts, considered in this study. A real-life application based on the smartphone accelerometer data-set, for the implementation of the proposed scheme, is also presented.

## 1. Introduction

Statistical process control (SPC) is a collection of tools for the monitoring of process parameters. The most valuable of these tools is control chart (cf. Montgomery [1]). Shewhart, cumulative sum (CUSUM) and exponentially weighted moving average (EWMA) charts (cf. [2–4]) are the commonly used control chart structures to monitor the parameters of the process. The simplicity and ease of interpretation make Shewhart charts more common in use, but they are relatively insensitive to small shifts in process parameters, whereas, CUSUM and EWMA control charts are mostly used for the detection of smaller shifts in process parameters (cf. [1]).

In parametric control charts, the parent distribution of the process production is usually known and commonly assumed to be a normal. If the distribution of the process production is unknown, the traditional control limits no longer remain effective and the detection ability of parametric control charts can be negatively affected. This leads us to the development of control charts that are not specifically designed under the assumption of normality or any other parametric distribution. In SPC literature, the nonparametric control charts are widely employed and have numerous advantages for the monitoring of real processes (cf. Chakraborti et al. [5]). For recent literature on nonparametric charts, the interested readers may go through the contributions by [6–14].

In SPC literature, various sampling techniques are used to improve the performance of the parametric and nonparametric control charts. Of these, simple random sampling (SRS), (cf. Montgomery [1]), double sampling (DS) (cf. Croasdale [15]), ranked set sampling (RSS) and its different forms (cf. [16–17]), repetitive sampling (RS) (cf. [18–19]) and variable sampling interval (VSI) (cf. [20–21]) are famous ones. Balamurali and Jun [22] showed that the RS scheme is more efficient than single and double sampling schemes but it is not better than the sequential sampling (SS) scheme. The SS was introduced by Wald [23] as a tool for more effective industrial quality control during second world war. The SS is a sampling plan in which an undetermined number of samples are tested one by one, accumulating the results, until a decision can be made. In SS the sample size i.e., *n* is not fixed in advanced. Balamurali and Jun [22] mentioned that the SS is more efficient as compared to DS procedure. The SS and RS schemes are quite similar to each other. Both sampling schemes have a similar pair of limits and decision criteria is same for both designs. The only difference exists between these two designs when a sample falls in the no-decision interval. In RS, the sampler discards the sample that falls in the no-decision interval and the resampling will continue until a decision is reached. On the other hand, in SS, the sampler doesn’t ignore the sample that falls in the no-decision interval, the sampler draws a new sample and update the information with previous sample, until sample statistic falls in either of the decisive zones.

By exploring the literature, we found that no study as of yet, utilizes the SS scheme for increasing the efficiency of the nonparametric control charts. To fill this gap, we propose a nonparametric EWMA sign chart, based on arcsine transformation, using the SS scheme, for efficient monitoring of process location. The rest of the article is as follows: the description of the existing and proposed charts is presented in Section 2. The performance comparisons are provided in Section 3. A real application of the proposed chart is given in Section 4. Finally, the summary and conclusions are provided in Section 5.

## 2. Description of nonparametric control charts

In this section, we provide a brief description of some useful non-parametric charts such as: the nonparametric EWMA sign (EWMA-Sign), the arcsine EWMA (AEWMA-Sign) charts, proposed by Yang et al. [24], and the nonparametric CUSUM sign (CUSUM-Sign) chart proposed by Yang and Cheng [25].

### 2.1. EWMA-Sign chart

Let *X* be the variable of interest with mean value *θ* and *T* = *X*−*θ* defines the respective deviations from its mean value. Let *p* denote the proportion of positive deviations i.e. *p* = *P*(*T*>0). For in-control process, *p* = 0.5 and for out-of-control process, *p* = *p*_1_≠0.5. The sign test statistic is written as:

$$T^{+}=\sum_{j=1}^{n}I(X_{j}-\theta >0),$$ (1)

where *I*(.)is given as:

$$I(X_{j}-\theta >0)=\left\{ \begin{array}{l} 1,\;if\;T^{+}=(X_{j}-\theta >0)\\ 0,\;\mathrm{otherwise} \end{array}.\right.$$

where *j* = 1,2,…,*n*.

Koti and Babu [26] showed that *T*^+^ follows the binomial distribution with parameters *n* and *p*. Moreover, *E*(*T*^+^) = *n*/2 and *Var*(*T*^+^) = *n*/4, respectively. The EWMA statistic based on (1) is written as:

$${EWMA}_{T_{i}^{+}}=\lambda T_{i}^{+}+(1-\lambda ){EWMA}_{T_{i-1}^{+}}$$ (2)

where *λ* is the smoothing parameter ranging from 0 to 1.

Yang et al. [24] proposed the EWMA-Sign chart to monitor the process target. The mean and variance of the EWMA statistic in (2) are respectively given as (Abbasi [27] and Yang et al. [24]):

$$E({EWMA}_{T_{i}^{+}})=n/2\;and\;Var({EWMA}_{T_{i}^{+}})=\frac{\lambda }{2-\lambda }\left(\frac{n}{4}\right).$$

The asymptotic control limits of Yang et al. [24] chart are

$${UCL}_{{EWMA}_{T^{+}}}=\frac{n}{2}+L\sqrt{\frac{\lambda }{2-\lambda }(\frac{n}{4})},$$

$${CL}_{{EWMA}_{T^{+}}}=\frac{n}{2},$$ (3)

$${LCL}_{{EWMA}_{T^{+}}}=\frac{n}{2}-L\sqrt{\frac{\lambda }{2-\lambda }(\frac{n}{4})}.$$

where *L* is the width of the control limits.

### 2.2. AEWMA-Sign chart

Yang et al. [24] observed that due to the asymmetric behavior of the binomial distribution for small to moderate sample size *n*, the in-control average run length (*ARL*_0_) values of the EWMA sign chart are not equal to the usually known value of 370 when *p* = 0.5. So to overcome this deficiency, Yang et al. [24] applied the arcsine transformation i.e., $T={sin}^{-1}(\sqrt{p})$. The distribution of *T* under the arcsine transformation follows the normal distribution with mean ${sin}^{-1}(\sqrt{p})$ and variance $\left(\frac{1}{4n}\right)$. The EWMA statistic based on the arcsine transformation is defined as:

$${EWMA}_{T_{i}}=\lambda T_{i}+(1-\lambda ){EWMA}_{T_{i-1}}$$ (4)

The starting value of ${EWMA}_{T_{i}}$ is set as the mean value of *T* as ${EWMA}_{T_{0}}={sin}^{-1}(\sqrt{0.5})$. The mean and variance of the ${EWMA}_{T_{i}}$ are $E({EWMA}_{T_{i}})={sin}^{-1}(\sqrt{0.5})$ and $Var({EWMA}_{T_{i}})=\frac{\lambda }{2-\lambda }\left(\frac{1}{4n}\right)$, respectively (cf. Yang et al. [24]).

So, the control limits of the arcsine EWMA sign chart are:

$${UCL}_{{EWMA}_{T}}={sin}^{-1}(\sqrt{0.5})+L\sqrt{\frac{\lambda }{2-\lambda }(\frac{1}{4n})},$$

$${CL}_{{EWMA}_{T}}={sin}^{-1}(\sqrt{0.5}),$$ (5)

$${LCL}_{{EWMA}_{T}}={sin}^{-1}(\sqrt{0.5})-L\sqrt{\frac{\lambda }{2-\lambda }(\frac{1}{4n})}.$$

where *p* = 0.5 represents the in-control state of the process. If any ${EWMA}_{T}\geq {UCL}_{{EWMA}_{T}}$ or ${EWMA}_{T}\leq {LCL}_{{EWMA}_{T}}$, the process is considered to be out-of-control. The AEWMA-Sign chart shows slightly better shift detection ability as compared to the EWMA-Sign chart (cf. Yang et al. [24]).

### 2.3. CUSUM-Sign chart

Using the statistic given in (1), Yang and Cheng [25] developed the two plotting statistic i.e., $C_{t}^{+}$ and $C_{t}^{-}$ of the CUSUM sign chart as follows:

$$\begin{array}{c} C_{t}^{+}=max(0,C_{t-1}^{+}+T_{t}^{+}-(np_{0}+k))\\ C_{t}^{-}=min(0,C_{t-1}^{-}-(np_{0}-k)+T_{t}^{+}) \end{array}\}$$ (6)

where *t* = 1,2,… and initially, $C_{t}^{+}=0$ and $C_{t}^{-}=0.$ The statistics given in (6) are plotted against their control limits *h* and −*h*, respectively. The process is considered to be out-of-control if $C_{t}^{+}\geq h$ or $C_{t}^{-}\leq -h$, else, it is in-control. For *k* = 0.5, *h* = 10.65 and *n* = 10, the *ARL*_0_ of the CUSUM−Sign chart is 370.

### 2.4. Proposed arcsine EWMA sign chart

In this section, we combine the idea of SS scheme with the nonparametric arcsine EWMA sign chart, namely the SAEWMA-Sign chart. The SS scheme is more economical and time-saving in comparison to the RS and DS schemes. In SS scheme undetermined number of samples are tested one by one, adding the results until a decision can be made. The construction of the SAEWMA-Sign chart is based on the following two steps:

**Step I:** A sample of size *n* is selected for the computation of the EWMA statistics, using the expression given in (4).

**Step II:** The SAEWMA-Sign chart has two pairs of control limits which consist of two upper control limits i.e., *UCL*_1_ and *UCL*_2_ and two lower control limits i.e., *LCL*_1_ and *LCL*_2_. The four control limits of the proposed chart, based on SS scheme are given as follows (cf. Aslam et al. [19]):

$$\begin{array}{c} {UCL}_{1}={sin}^{-1}(\sqrt{0.5})+L_{1}\sqrt{\frac{\lambda }{2-\lambda }(\frac{1}{4n})}\\ {LCL}_{1}={sin}^{-1}(\sqrt{0.5})-L_{1}\sqrt{\frac{\lambda }{2-\lambda }(\frac{1}{4n})}\\ {UCL}_{2}={sin}^{-1}(\sqrt{0.5})+L_{2}\sqrt{\frac{\lambda }{2-\lambda }(\frac{1}{4n})}\\ {UCL}_{2}={sin}^{-1}(\sqrt{0.5})-L_{2}\sqrt{\frac{\lambda }{2-\lambda }(\frac{1}{4n})} \end{array}\}$$ (7)

In (7), *L*_1_ and *L*_2_ (*L*_1_≥*L*_2_) are the two control limits coefficients to be determined.

**Fig 1:**
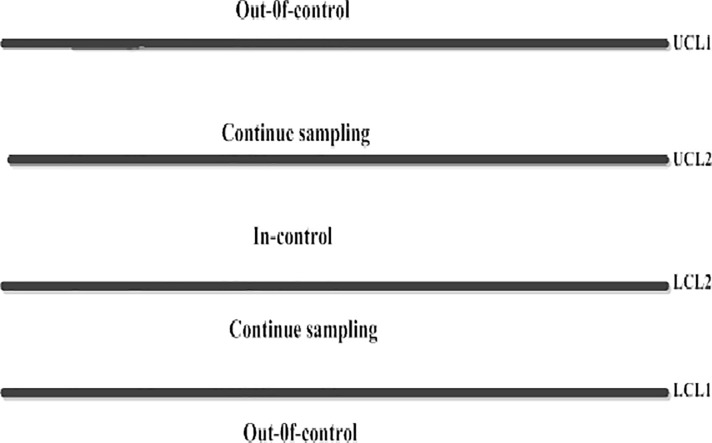
Decision criteria of the proposed chart (A model display).

The decision criteria of SAEWMA-Sign chart is outlined as:

- the process is stated as out-of-control if ${EWMA}_{T_{i}}\geq {UCL}_{1}$ or ${EWMA}_{T_{i}}\leq {LCL}_{1}$;
- if ${LCL}_{2}\leq {EWMA}_{T_{i}}\leq {UCL}_{2}$ the process is declared to be in-control;
- if ${LCL}_{1}\leq {EWMA}_{T_{i}}\leq {LCL}_{2}$ or ${UCL}_{2}\leq {EWMA}_{T_{i}}\leq {UCL}_{1}$ then continue sampling and go to step I (cf. Fig 1).

***Special Case*:** If *L*_1_ = *L*_2_, then the proposed scheme is similar to the AEWMA-Sign chart under the SRS scheme. So, the proposed chart is a special case of the chart proposed by Yang et al. [24].

## 3. Performance assessment

There are a variety of measures that can be used to evaluate the performance of control charts. Some of the important measures, used in this study are:

**Average run length (*ARL*)** is broadly used by the researchers to assess the performance of control charts. The in-control and out-of-control *ARLs* are denoted by *ARL*_0_ and *ARL*_1_, respectively. Some researchers recommend the use of standard deviation run length (*SDRL*) and median run length (*MDRL*), due to the skewed behavior of the run length (RL) distribution.

The *ARL*, *MDRL* and *SDRL* are defined as:

$$ARL=\frac{\sum_{m}{\left(RL\right)}_{m}}{m},$$ (8)

MDRL=Median(RL), (9)

$$SDRL=\sqrt{E{\left(RL\right)}^{2}-{\left(E(RL)\right)}^{2}}.$$ (10)

We have adopted Monte Carlo (MC) simulations based on 5×10^4^ iterations to find the results. The advantages of MC simulation over the other methods can be seen in Dyer [28].

The computational algorithms for the computation of different run length measures is described below: described below:

- Generate a random sample of size *n* from the binomial distribution, having parameters *n* and *p* = *p*_0_ = 0.5, call it *T*_*i*_.
- Compute the ${EWMA}_{T_{i}}$ statistics using the expression given in (4).
- For a fixed level of *λ*, select values for *L*_*1*_ and *L*_*2*_ for the computation of control limits in (6), for a pre-specified *ARL*_0_.
- The sample number at which the plotting statistic falls outside the *UCL*_1_ or *LCL*_1_ is called a run length. If ${LCL}_{1}\leq {EWMA}_{T_{i}}\leq {LCL}_{2}$ or ${UCL}_{2}\leq {EWMA}_{T_{i}}\leq {UCL}_{1}$, we continue resampling and repeat steps (i)–(iii) unless the plotting statistics falls in either of the decisive zones.
- Repeat steps (i)-(v) 5×10^4^ times to compute the in-control *ARL* as the mean of these run lengths.

For the out-of-control ARL, shifts are introduced by generating random observations from Binomial distribution using parameters *n* and *p* = *p*_1_≠0.5. To evaluate the performance of the proposed chart, we chose various combination of *L*_1_, *L*_2_, *n* and *λ*, to achieve a pre-specified *ARL*_0_. It is to be mentioned that the design parameter *L*_2_ is obtained by using the formula *L*_2_ = *L*−φ*L where *L* is defined earlier in Section 2 and *φ* helps in defining the non-decisive zone. For our study purpose, we used *λ* = 0.05 and 0.25 for the proposed chart and found the control chart multipliers *L*_*1*_ and *L*_*2*_ for fixing *ARL*_0_ = 370. Moreover, we have used *φ* = 0.02(0.02)0.1 in this study.

For these design parameters, we have obtained the run length properties of the proposed chart such as ARL, MDRL and SDRL. These results are provided in Table 1. From Table 1, we advocate the following interesting points:

- The *ARL*_0_ values are close to the desired value of 370 when the value of *p* = 0.5 (for example for *λ* = 0.05,*L*_1_ = 2.665,*L*_2_ = 2.619,*φ* = 0.02,*ARL*_0_ = 369 and for *λ* = 0.25, *L*_1_ = 3.271,*L*_2_ = 3.155,*φ* = 0.02, *ARL*_0_ = 370).
- It is noted that the efficiency of the proposed chart to detect small shifts in the process location, increases as the value of *λ* decreases (for example for *λ* = 0.25,*L*_1_ = 3.362,*L*_2_ = 3.090,*φ* = 0.04,*p*_1_ = 0.51, *ARL*_1_ = 303 and for *λ* = 0.05, *L*_1_ = 2.667,*L*_2_ = 2.565,*φ* = 0.04,*p*_1_ = 0.51,*ARL*_1_ = 257).
- It is observed that the shift detection ability of the proposed scheme increases as the value of *φ* increase (for example for *λ* = 0.05, *L*_1_ = 2.665,*L*_2_ = 2.619,*p*_1_ = 0.51,*φ* = 0.02,*ARL*_1_ = 272 and for *λ* = 0.05, *L*_1_ = 2.665,*L*_2_ = 2.619,*p*_1_ = 0.51,*φ* = 0.08, *ARL*_1_ = 228).
- The values of *MDRL* and *SDRL* decreases as the value of *φ* increases (for example for *λ* = 0.05,*p*_1_ = 0.52,*φ* = 0.02, *MDRL* = 120, *SDRL* = 150, and for *λ* = 0.05, *p*_1_ = 0.52, *φ* = 0.1,*MDRL* = 93,*SDRL* = 112).
- The *MDRL* and *SDRL* also decreases with an increase in the level of *p*_1_, considering fixed *λ* and *φ*.

**Table 1:** Run length properties of the proposed chart under *ARL*_0_≈370.

*p*_1_	*φ*	*λ* = 0.05, *n* = 10					*λ* = 0.25, *n* = 10				
		0.02	0.04	0.06	0.08	0.1	0.02	0.04	0.06	0.08	0.1
	*L*_1_	2.665	2.667	2.679	2.693	2.74	3.271	3.362	3.492	3.774	7.514
	*L*_2_	2.619	2.565	2.512	2.458	2.405	3.155	3.09	3.026	2.961	2.897
0.5	*ARL*	369	369	369	371	370	370	371	369	369	367
	*MDRL*	260	259	259	259	259	255	257	257	255	249
	*SDRL*	359	360	358	363	365	372	375	373	374	369
0.51	*ARL*	272	257	243	228	214	323	303	285	267	246
	*MDRL*	191	181	173	164	153	223	209	198	187	172
	*SDRL*	257	244	231	212	201	320	300	283	264	245
0.52	*ARL*	166	154	145	135	126	252	227	209	189	169
	*MDRL*	120	113	107	99	93	175	160	146	132	117
	*SDRL*	150	138	129	120	112	247	223	206	188	169
0.53	*ARL*	103	97	92	86	82	184	165	149	133	120
	*MDRL*	77	73	69	65	61	130	116	105	94	86
	*SDRL*	88	83	77	72	68	178	160	145	129	116
0.54	*ARL*	69	66	63	59	56	134	121	108	98	88
	*MDRL*	53	51	48	46	44	95	87	77	70	64
	*SDRL*	55	52	49	46	44	130	117	106	93	83
0.55	*ARL*	50	48	46	44	42	98	89	80	72	65
	*MDRL*	40	38	37	35	33	70	64	57	51	47
	*SDRL*	36	35	34	32	30	94	84	76	67	60
0.6	*ARL*	19	18	18	17	17	27	25	23	22	20
	*MDRL*	17	16	16	15	15	20	19	17	16	15
	*SDRL*	9	9	9	9	9	23	21	20	18	17
0.7	*ARL*	8	8	7	7	7	7	7	6	6	6
	*MDRL*	7	7	7	7	7	6	6	5	5	5
	*SDRL*	3	3	3	2	2	4	4	4	3	3
0.85	*ARL*	4	4	4	4	4	3	3	3	2	2
	*MDRL*	4	4	4	4	4	3	3	3	3	2
	*SDRL*	1	1	1	1	1	1	1	1	1	1
0.95	*ARL*	3	3	3	3	2	1	1	1	1	1
	*MDRL*	3	3	3	3	2	1	1	1	1	1
	*SDRL*	1	1	1	1	1	1	1	1	1	1

**Fig 2:**
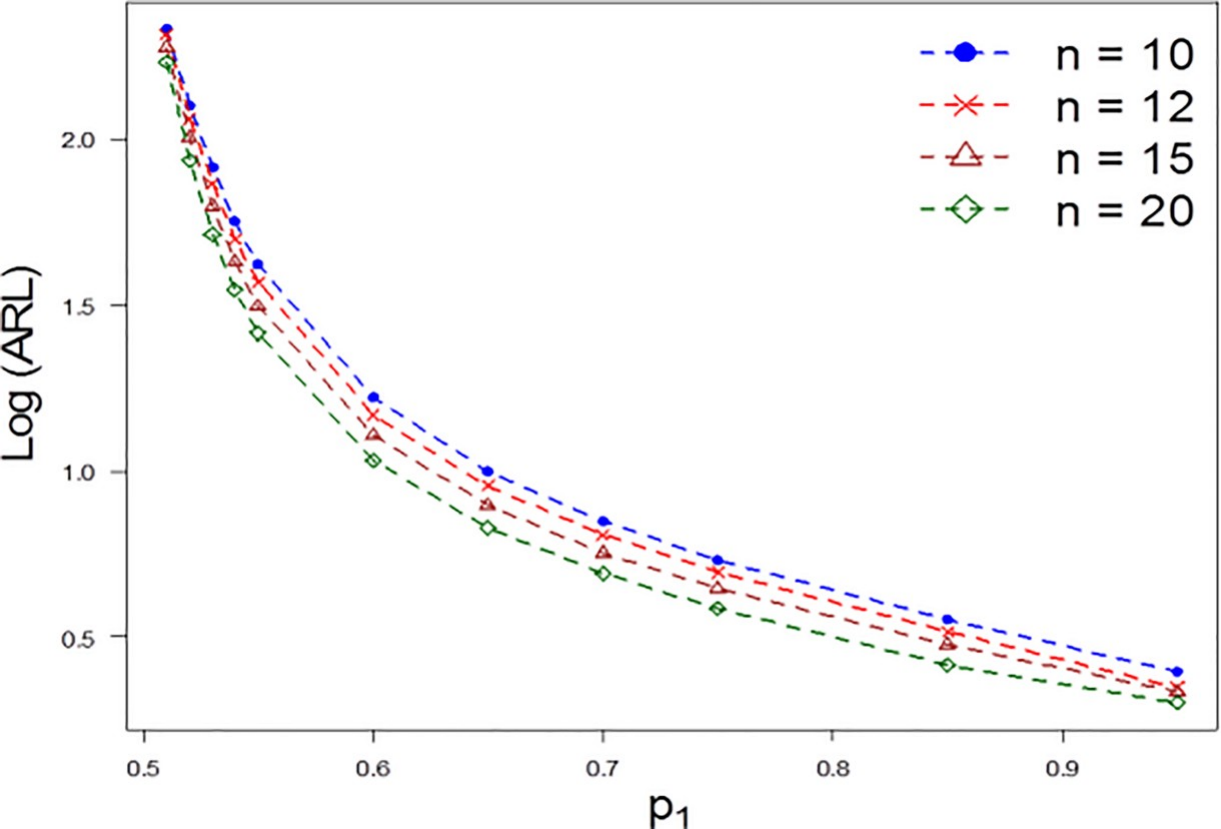
*ARL* comparison of the proposed chart for different levels of *n* when *λ* = 0.05 and *φ* = 0.1.

**Fig 3:**
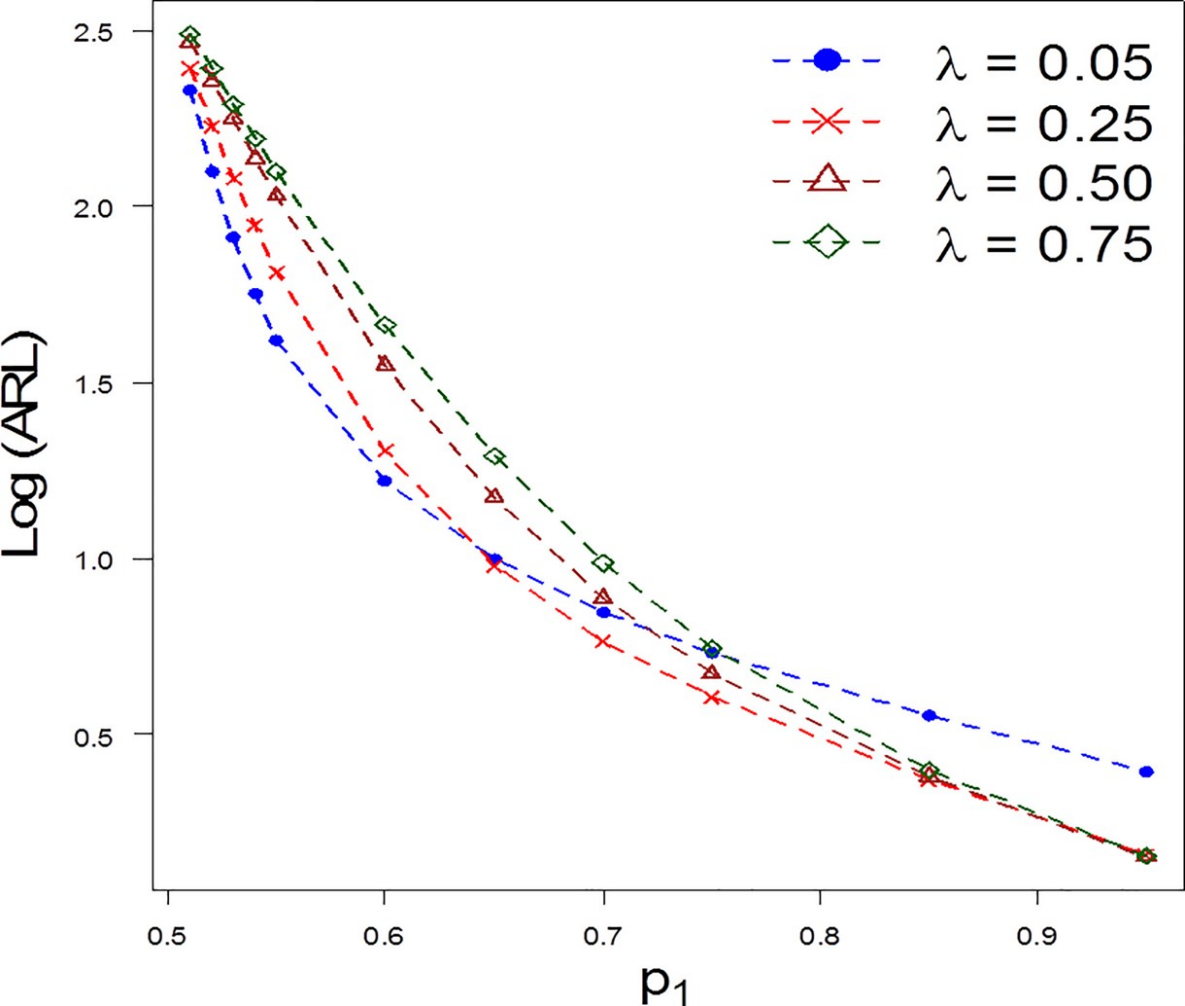
*ARL* comparison of the proposed chart for different levels of *λ* when *n* = 10 and *φ* = 0.1.

To get more insight of the run length distribution for the proposed chart, we also computed the run length properties at varying levels of *n* and *λ*. As the value of the *n* increases, the detection ability of the proposed chart increases. For example, for *n* = 10,*p*_1_ = 0.55,*ARL*_1_ = 42 and *n* = 15, *p*_1_ = 0.55,*ARL*_1_ = 31 (cf. Table 2 and Fig 2). On the other hand, as the value of *λ* increases the shift detection ability of the proposed chart decreases. For example, for *λ* = 0.05,*p*_1_ = 0.6,*ARL*_1_ = 17 and *λ* = 0.5, *p*_1_ = 0.6,*ARL*_1_ = 35 (cf. Table 3 and Fig 3).

**Table 2:** Run length properties of the proposed chart for different levels of *n* when *λ* = 0.05 and *φ* = 0.1.

*p*_1_	*n*	10	12	15	20
	*L*_1_	2.740	2.678	2.652	2.633
	*L*_2_	2.405	2.369	2.328	2.300
0.5	*ARL*	369.9	369	370.9	370
	*MDRL*	259	264	266	262
	*SDRL*	364	351	357	352
0.51	*ARL*	214	205	187	169
	*MDRL*	153	145	132	120
	*SDRL*	201	192	174	156
0.52	*ARL*	126	114	100	86
	*MDRL*	93	85	74	65
	*SDRL*	112	99	86	72
0.53	*ARL*	81	72	62	51
	*MDRL*	61	55	48	40
	*SDRL*	68	60	50	39
0.54	*ARL*	56	50	43	35
	*MDRL*	44	39	34	28
	*SDRL*	44	38	31	24
0.55	*ARL*	42	37	31	26
	*MDRL*	33	30	26	22
	*SDRL*	30	26	21	16
0.6	*ARL*	17	15	13	11
	*MDRL*	15	13	12	10
	*SDRL*	81	7	6	4
0.7	*ARL*	7	6	6	5
	*MDRL*	7	6	5	5
	*SDRL*	2	2	2	1
0.85	*ARL*	4	3	3	3
	*MDRL*	4	3	3	3
	*SDRL*	1	1	1	1
0.95	*ARL*	2	2	2	2
	*MDRL*	2	2	2	2
	*SDRL*	1	0	0	0

**Table 3:** Run length properties of the proposed chart for different levels of *λ* when *n* = 10 and *φ* = 0.1.

*p*_1_	*λ*	0.05	0.25	0.5	0.75
	*L*_1_	2.740	7.514	4.732	10
	*L*_2_	2.405	2.897	3.098	2.979
0.5	*ARL*	370	367	371	245
	*MDRL*	259	249	257	169
	*SDRL*	365	369	372	244
0.51	*ARL*	214	246	293	197
	*MDRL*	153	172	203	137
	*SDRL*	201	245	293	199
0.52	*ARL*	126	169	227	159
	*MDRL*	93	117	156	111
	*SDRL*	112	169	230	160
0.53	*ARL*	82	120	177	129
	*MDRL*	61	86	123	90
	*SDRL*	68	116	179	129
0.54	*ARL*	56	88	137	106
	*MDRL*	44	64	95	74
	*SDRL*	44	83	137	105
0.55	*ARL*	42	65	107	86
	*MDRL*	33	47	75	59
	*SDRL*	30	60	105	86
0.6	*ARL*	17	20	35	34
	*MDRL*	15	15	25	24
	*SDRL*	9	17	33	33
0.7	*ARL*	7	6	8	8
	*MDRL*	7	5	6	6
	*SDRL*	2	3	6	7
0.85	*ARL*	2	1	1	1
	*MDRL*	2	1	1	1
	*SDRL*	1	1	1	1
0.95	*ARL*	2	1	1	1
	*MDRL*	2	1	1	1
	*SDRL*	1	1	1	1

### 3.1. Comparative analysis

In this section, we present a comparison of the proposed scheme with the EWMA-Sign chart, the AEWMA-Sign chart and the CUSUM-Sign chart. To make valid comparisons with existing counterparts, *ARL*_0_ of all selected charts is fixed at a pre-specified level i.e., *ARL*_0_ = 370.

#### 3.1.1. Proposed Vs EWMA-Sign

The *ARL* values of the EWMA-Sign chart and proposed SAEWMA-sign chart are presented in Table 4, under different shift levels. The comparison reveals that SAEWMA-Sign chart performs efficiently at different shift levels (for example, with *n* = 10, *p*_1_ = 0.51,0.53,0.55 and 0.7, the ARL values of the proposed SAEWMA-Sign chart are *ARL*_1_ = 214,82,42,7 whereas the corresponding *ARL*_1_ = 288,106,52,8 for EWMA-Sign chart (cf. Table 4)). From Table 4, it is revealed that for all the choices of *n* and *p*_1_ the proposed SAEWMA-Sign chart performs more efficiently relative to the EWMA-Sign chart. These results show that the proposed SAEWMA-Sign chart is far better than EWMA-Sign chart in terms of detecting all levels of shifts.

**Table 4:** *ARL* values of the proposed and existing control charts when λ = 0.05 for different levels of *n*.

*n*	*Charts*	*Profiles*	*p*_1_									
			0.5	0.51	0.52	0.53	0.54	0.55	0.6	0.7	0.85	0.95
10	*Proposed*	*ARL*	370	214	126	82	56	42	17	7	4	2
		*MDRL*	259	153	93	62	44	33	15	7	4	2
		*SDRL*	365	201	112	69	44	30	9	2	1	1
	*AEWMA*−*Sign*	*ARL*	369	288	174	106	72	52	19	8	4	3
		*MDRL*	253	204	126	80	55	41	17	8	4	3
		*SDRL*	357	272	159	90	56	38	10	3	1	1
	*EWMA*−*Sign*	*ARL*	371	292	171	108	70	51	19	8	4	3
		*MDRL*	261	205	126	79	54	41	17	8	4	3
		*SDRL*	362	277	154	92	54	37	9	2	1	1
	*CUSUM*−*Sign*	*ARL*	376	317	216	138	90	63	20	8	4	3
		*MDRL*	260	221	155	99	67	48	18	8	4	3
		*SDRL*	372	304	203	125	77	52	11	3	1	0
15	*Proposed*	*ARL*	371	188	100	63	43	31	13	6	3	2
		*MDRL*	266	132	74	48	34	26	12	5	3	2
		*SDRL*	357	175	86	50	33	21	6	2	1	0
	*AEWMA*−*Sign*	*ARL*	369	255	138	81	53	38	15	6	3	2
		*MDRL*	261	186	101	62	43	31	13	6	3	2
		*SDRL*	354	250	122	67	39	25	6	2	1	0
	*EWMA*−*Sign*	*ARL*	369	256	140	82	53	38	15	6	4	3
		*MDRL*	258	183	102	63	43	32	13	6	4	3
		*SDRL*	350	239	124	66	38	25	6	2	1	0
	*CUSUM*−*Sign*	*ARL*	368	303	193	117	73	49	15	6	3	2
		*MDRL*	270	215	137	85	54	37	13	5	3	2
		*SDRL*	384	293	182	107	65	40	8	2	1	0
20	*Proposed*	*ARL*	370	169	86	51	35	26	11	5	3	2
		*MDRL*	262	120	65	40	28	22	10	5	3	2
		*SDRL*	352	156	73	39	24	16	4	1	1	0
	*AEWMA*−*Sign*	*ARL*	368	234	115	66	43	31	12	5	3	2
		*MDRL*	259	168	86	51	35	26	11	5	3	2
		*SDRL*	357	221	101	51	29	19	5	1	1	0
	*EWMA*−*Sign*	*ARL*	370	235	116	66	43	31	12	6	3	3
		*MDRL*	262	167	86	51	35	26	11	5	3	3
		*SDRL*	355	221	100	52	29	19	5	1	0	0
	*CUSUM*−*Sign*	*ARL*	358	286	173	98	61	40	12	5	2	2
		*MDRL*	254	202	122	71	45	30	10	4	2	2
		*SDRL*	345	276	165	89	53	33	6	1	1	0

#### 3.1.2. Proposed Vs AEWMA-Sign

The *ARL* values of the AEWMA-Sign chart and proposed chart are compared in Table 4 at various kinds of shifts. Based on Table 4, we observed that the proposed SAEWMA-Sign chart has significantly better performance as compared to the AEWMA-Sign chart, (for example, with *n* = 15, *p*_1_ = 0.51,0.53,0.55 and 0.7 the ARL of proposed SAEWMA-Sign chart are *ARL*_1_ = 188,63,31,6 against *ARL*_1_ = 255,81,38,6 for the AEWMA-Sign chart). From these results, we noticed a significantly better performance of the SAEWMA-sign chart compared to AEWMA-sign chart.

#### 3.1.3. Proposed Vs CUSUM-Sign

The *ARL* values of the CUSUM-Sign chart are reported in Table 4. From Table 4, it is revealed that the *ARL*_1_ of the proposed SAEWMA-Sign chart is lower than CUSUM-Sign, under all shifts in the process location (for example, with *n* = 15, *p*_1_ = 0.51,0.53,0.55 and 0.7, of the ARLs of the proposed SAEWMA-Sign chart are *ARL*_1_ = 188,63,31,6 against ARL values of CUSUM-Sign chart are, which are *ARL*_1_ = 303,117,49,6). The above mentioned results clearly indicates that the superiority of the proposed SAEWMA-Sign chart against the CUSUM-Sign chart for all levels of shifts.

### 3.2. A comparative analysis among single, double, repetitive and sequential sampling based charting schemes

In this section, we present a comparison of various charting schemes based on single, double, repetitive and sequential sampling mechanisms. We have evaluated the performance of all of these schemes in term of ARL, for varying shifts in location, by considering different widths of indecisive zones for some useful combinations of *φ* and *p*_1_ (cf. Table 5). In order to strengthen the findings of our comparative analysis, we have also computed the results of the average number of samples used (for single, double, repetitive and sequential schemes) for in-control and out-of-control processes. These results are reported in Table 6 for the same combinations of *φ* and *p*_1_, as used for Table 5.

**Table 5:** *ARL* values of single, DS, RS and SS schemes when *λ* = 0.05 and *n* = 10.

*φ*	*Sampling* *Schemes*	*p*_1_									
		0.5	0.51	0.52	0.53	0.54	0.55	0.6	0.7	0.85	0.95
0.02	*Single*	369	289	178	110	73	52	19	8	4	3
	*DS*	369	272	164	104	69	50	19	8	4	3
	*RS*	369	292	181	112	74	53	19	8	4	3
	*SS*	369	272	166	103	69	50	19	8	4	3
0.06	*Single*	369	289	178	110	73	52	19	8	4	3
	*DS*	371	245	147	92	64	46	18	7	4	3
	*RS*	369	292	182	112	75	54	19	8	4	3
	*SS*	369	243	145	91	63	46	18	7	4	3
0.1	*Single*	369	289	178	110	73	52	19	8	4	3
	*DS*	374	221	128	80	56	42	17	7	4	2
	*RS*	370	298	186	115	77	55	19	8	4	3
	*SS*	370	214	126	79	56	42	17	7	4	2

**Table 6:** Average number of samples in the indecisive region for DS, RS and SS at *λ* = 0.05 and *n* = 10.

*φ*	*Sampling* *Schemes*	*p*_1_									
		0.5	0.51	0.52	0.53	0.54	0.55	0.6	0.7	0.85	0.95
0.02	*DS*	0.127	0.187	0.203	0.202	0.206	0.194	0.167	0.115	0.064	0.003
	*RS*	0.204	0.203	0.200	0.203	0.183	0.176	0.161	0.104	0.067	0.004
	*SS*	0.247	0.224	0.217	0.201	0.200	0.198	0.175	0.113	0.067	0.004
0.04	*DS*	0.401	0.528	0.555	0.550	0.542	0.525	0.474	0.365	0.178	0.038
	*RS*	0.657	0.638	0.612	0.593	0.571	0.562	0.456	0.297	0.059	0.002
	*SS*	0.834	0.646	0.576	0.564	0.547	0.536	0.465	0.356	0.174	0.034
0.1	*DS*	0.715	0.805	0.822	0.820	0.814	0.804	0.749	0.636	0.414	0.243
	*RS*	1.242	1.238	1.167	1.108	1.063	0.998	0.814	0.516	0.262	0.487
	*SS*	1.519	1.024	0.875	0.831	0.817	0.802	0.759	0.639	0.420	0.235

The comparative analysis reveals the following:

- The SS scheme detects the shifts more efficiently as compared to the single, DS and RS schemes. For instance, at *p*_1_ = 0.51 and *φ* = 0.1 the *ARL*_1_ values are 289, 221, 298 and 214 for single, DS, RS and SS schemes, respectively, as may be seen in Table 5.
- The average sample size for DS, RS and SS schemes (cf. Table 6) reveals that the SS scheme gains an edge over other scheme (for almost all shifts levels) with a marginal increase in the average number of samples used (cf. Table 5). For instance, if *p*_1_ = 0.52 and *φ* = 0.1, the average number of samples used are 10.822, 11.167 and 10.875 for DS, RS and SS schemes respectively (cf. Table 6).
- The performance of the DS and SS schemes are in close competition, especially when *p*_1_>0.54 (cf. Table 5). The performance of the SS scheme is relatively better than the single, DS and RS schemes when 0.51≤*p*_1_≤0.54 (cf. Table 5).
- With an increase in the width of indecisive zone (i.e. *φ*), the SS scheme gets an advantage over others, followed by the superiority of DS. It is to be noted that RS scheme behaves in a reverse manner, the reason being ignoring the sample falling in indecisive zone. In real applications, having a wider indecisive zone may not be very practical, and hence we have chosen the indecisive regions of practical worth.

Therefore, we can say that the proposed chart based on SS scheme offers an efficient charting structure that is relatively better in detecting all levels of shifts, as compared to the existing control charts, considered in this study.

## 4. A real-life application on smartphone accelerometer data

In this section, we provide a real-life application of an accelerometer data-set for the proposed and the other schemes, considered in this study. An accelerometer is a device which has extensive variety of applications in various fields, such as to measure vibration on machines, cars, air blast pressure, earthquake and aftershocks etc. In this study, we have selected the smartphone accelerometer data-set for the monitoring purpose. This application presents the enactment of control charts for accelerometer data monitoring. We have selected total 50 subgroups of size 10 for this study (cf. Riaz et al. [29]). For the construction of the proposed and the AEWMA-Sign schemes, we used the following parameters, *λ* = 0.25, *L*_1_ = 3.492, *L*_2_ = 3.026, *P* = 0.06, *L* = 3.291 and *ARL*_0_≅370.

**Fig 4:**
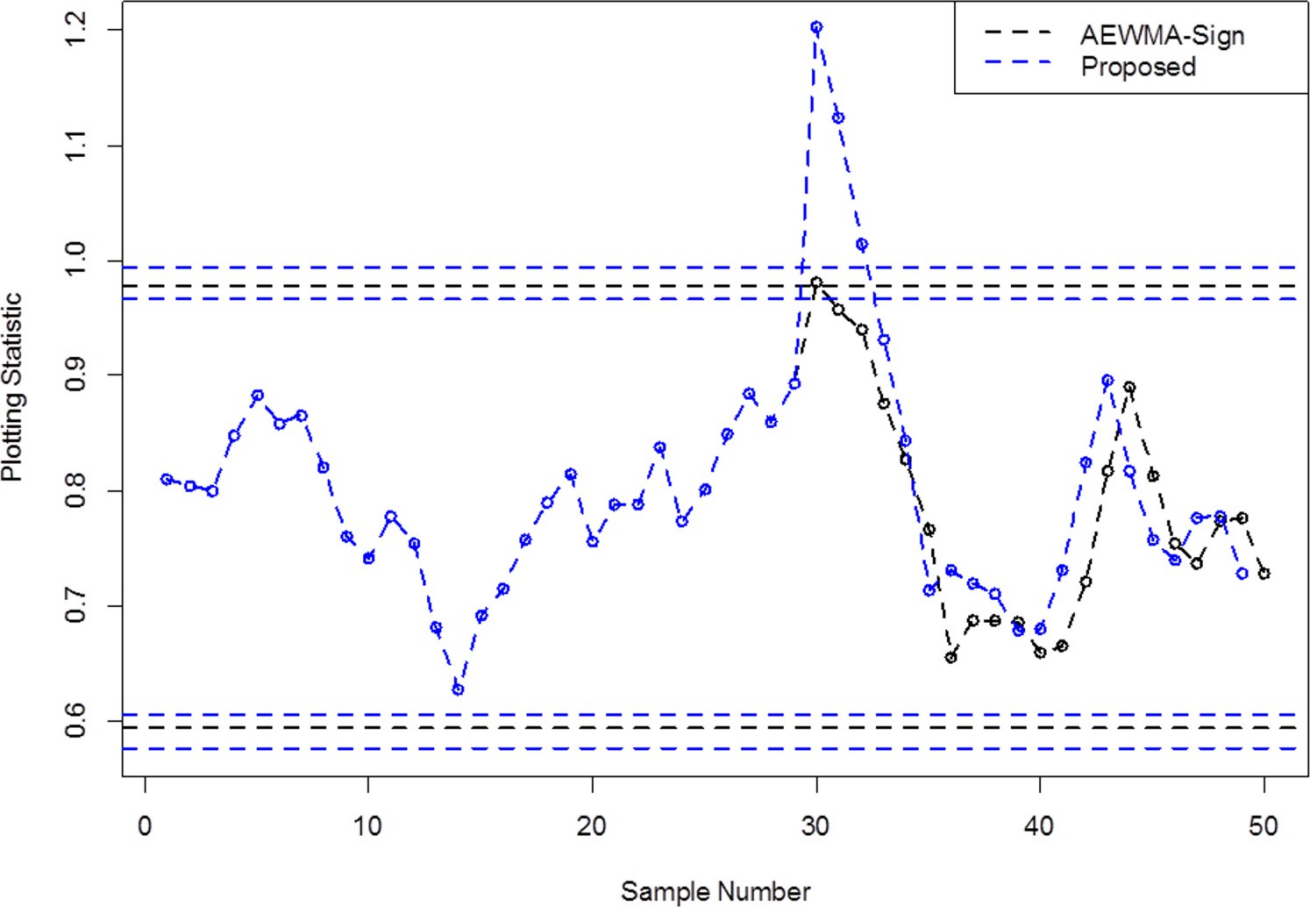
A real-life application using data-set of smartphone accelerometer.

The monitoring statistics given in (2) and (6) of the proposed SAEWMA-sign and the AEWMA-sign charts are thus constructed using the control limits given in (3) and (6), respectively. By observing the charts in Fig 4, following observations can be made for the smartphone accelerometer data-set:

- The AEWMA-Sign scheme proposed by Yang et al. [24] shows an out-of-control signal at sample # 30 (cf. Fig 4).
- The proposed SAEWMA chart based on the SS scheme offers three out-of- control signals at sample points 29, 30 and 31 (cf. Fig 4), which indicates the quick and better shift detection ability of the proposed scheme as compared to the AEWMA-Sign scheme.

We may conclude that the proposed scheme outshines the AEWMA-Sign scheme for detecting shifts in process location of the smartphone accelerometer data. The real life application also supported the finding in Section 3.

## 5. Summary, conclusions and recommendations

An efficient sampling strategy can be very effective in reducing the amount of waste produced by a process. Sequential sampling is one such mechanism. In this study, we have introduced a nonparametric arcsine EWMA sign chart, namely the SAEWMA-sign chart, based on the sequential sampling, in order to increase the detection ability of the arcsine EWMA sign chart. This performance analysis revealed that the proposed chart is an efficient chart that offers higher sensitivity to different types of changes in process parameters. It is also revealed that the proposed chart has quicker shift detection ability under all the design parameters as compared to the competing charts including EWMA-Sign, the AEWMA-Sign and the CUSUM-Sign charts. A real-life data set based on smartphone accelerometer is presented for the implementation of the proposed chart. The said application favors the new chart as a more beneficial statistical tool to detect abnormalities in process location.

The scope of this study may be extended to various other directions for future research such as memory charts for attributes and variables, single and multivariate quality characteristics of the process, under sequential sampling mechanism. Moreover, the proposed chart can be further investigated for parent skewed process distributions.

## Supporting information

S1 Dataset (DOCX)
