# Supplementary material for: An enhanced nonparametric EWMA sign control chart using sequential mechanism
Source: PLoS One. 2019 Nov 21;14(11):e0225330. doi: 10.1371/journal.pone.0225330 (PMC6872166; doi:10.1371/journal.pone.0225330)
Supplement: S1 Dataset — (DOCX) [file pone.0225330.s001.docx]

**S1 Dataset**

|  | X1 | X2 | X3 | X4 | X5 | X6 | X7 | X8 | X9 | X10 |
| --- | --- | --- | --- | --- | --- | --- | --- | --- | --- | --- |
| 1 | -6.5235 | -8.4600 | -7.2781 | -5.7618 | -7.0996 | -6.9175 | -6.7282 | -7.9327 | -5.7321 | -4.1027 |
| 2 | -7.6947 | -6.9865 | -7.0627 | -6.6176 | -6.5818 | -6.8365 | -6.0605 | -7.3459 | -6.9806 | -4.5788 |
| 3 | -8.3969 | -7.1662 | -7.3888 | -4.4490 | -7.6578 | -5.0406 | -5.1096 | -6.6152 | -6.0129 | -7.9030 |
| 4 | -8.2493 | -7.5185 | -6.6580 | -6.8175 | -5.5988 | -6.7961 | -6.8842 | -6.5378 | -7.3519 | -5.3702 |
| 5 | -7.7458 | -5.2322 | -6.6652 | -6.3831 | -6.1248 | -7.1531 | -6.2438 | -7.0520 | -6.8853 | -6.6818 |
| 6 | -7.0246 | -8.1826 | -7.7946 | -5.9868 | -7.6994 | -5.5071 | -6.0915 | -6.1177 | -7.0079 | -6.2914 |
| 7 | -6.6033 | -8.6885 | -5.8915 | -5.3095 | -8.4897 | -6.5009 | -5.7023 | -7.0817 | -7.7137 | -6.2593 |
| 8 | -7.2912 | -7.0770 | -6.6033 | -6.7937 | -5.5964 | -6.6854 | -7.0175 | -8.1553 | -7.9577 | -7.7423 |
| 9 | -7.1472 | -4.8192 | -7.9577 | -6.9568 | -6.9925 | -5.8320 | -7.7173 | -4.2848 | -8.1303 | -7.1817 |
| 10 | -6.2307 | -7.3138 | -6.5914 | -9.2455 | -8.1826 | -8.6801 | -6.8044 | -4.8763 | -7.8196 | -7.2769 |
| 11 | -8.1826 | -5.8701 | -8.5778 | -7.1448 | -4.6407 | -7.5007 | -6.0010 | -6.4176 | -6.7901 | -4.9751 |
| 12 | -6.4545 | -7.5507 | -7.4162 | -6.0986 | -9.1538 | -4.6109 | -8.9110 | -6.7925 | -6.9401 | -8.6302 |
| 13 | -5.4226 | -8.6837 | -7.5947 | -8.6432 | -7.4781 | -7.3578 | -7.0317 | -7.3531 | -6.6366 | -8.4278 |
| 14 | -7.3281 | -8.3886 | -5.7880 | -7.1924 | -7.9934 | -7.6661 | -6.9056 | -8.3743 | -8.9670 | -7.1353 |
| 15 | -5.0822 | -4.8846 | -7.1758 | -8.4540 | -5.4048 | -7.0163 | -6.4581 | -8.3100 | -6.6187 | -5.2988 |
| 16 | -6.0010 | -8.7111 | -9.1169 | -8.4957 | -6.7568 | -7.6554 | -5.9820 | -6.7592 | -7.2603 | -6.8532 |
| 17 | -8.0029 | -8.5016 | -5.9796 | -6.4640 | -8.7111 | -6.7878 | -4.6990 | -7.0175 | -6.5759 | -4.6728 |
| 18 | -8.4314 | -4.5347 | -5.5547 | -6.0641 | -4.1598 | -5.2453 | -8.4350 | -8.5826 | -8.1826 | -6.6068 |
| 19 | -7.4959 | -7.4400 | -5.7975 | -5.3500 | -4.8989 | -7.2162 | -5.8011 | -6.9865 | -5.8904 | -4.1277 |
| 20 | -6.9270 | -8.2433 | -7.0175 | -8.0220 | -6.9639 | -6.1308 | -8.2957 | -6.4259 | -6.0034 | -8.3897 |
| 21 | -8.4040 | -7.5173 | -8.5457 | -6.0379 | -4.5264 | -5.9844 | -4.4597 | -5.3976 | -5.7523 | -8.3040 |
| 22 | -7.6530 | -8.3981 | -4.6418 | -7.6804 | -4.6180 | -7.8649 | -7.7054 | -5.8094 | -5.9189 | -4.5443 |
| 23 | -5.1524 | -7.1400 | -7.7387 | -4.4919 | -7.7030 | -6.8532 | -6.3855 | -4.2443 | -6.0879 | -6.5426 |
| 24 | -8.4504 | -7.5602 | -6.2046 | -7.1698 | -6.9294 | -7.6328 | -8.4909 | -6.9199 | -6.1474 | -6.1236 |
| 25 | -8.0041 | -6.9544 | -6.8937 | -6.3688 | -4.7525 | -6.8389 | -7.1377 | -6.6652 | -6.1986 | -7.7720 |
| 26 | -6.2653 | -7.7673 | -6.9151 | -4.1408 | -4.6097 | -6.2688 | -6.6652 | -8.5076 | -5.5226 | -7.1722 |
| 27 | -6.4295 | -7.2150 | -7.5185 | -4.5407 | -4.1789 | -7.1139 | -4.4288 | -4.2431 | -6.6104 | -6.1153 |
| 28 | -8.7551 | -7.2162 | -7.0067 | -6.7937 | -7.0044 | -5.5333 | -5.3369 | -6.7223 | -7.5209 | -6.5795 |
| 29 | -8.5647 | -6.8651 | -5.2822 | -4.0479 | -6.0605 | -5.2881 | -6.3688 | -8.1350 | -7.4126 | -5.8558 |
| 30 | -6.5366 | -5.2477 | -5.8975 | -7.3102 | -6.6366 | -4.0694 | -6.0558 | -4.6121 | -6.5366 | -6.7901 |
| 31 | -8.5492 | -4.4336 | -6.1760 | -9.2324 | -6.8663 | -6.5402 | -6.5426 | -8.6909 | -6.4640 | -7.9327 |
| 32 | -4.4074 | -6.8461 | -7.3186 | -6.1962 | -4.1872 | -6.9211 | -6.1927 | -8.4742 | -6.4712 | -7.1270 |
| 33 | -7.1877 | -8.3933 | -6.9187 | -8.0981 | -6.9056 | -6.9734 | -6.0260 | -6.8318 | -8.1743 | -8.4397 |
| 34 | -7.0877 | -7.6399 | -8.3528 | -6.5866 | -5.9237 | -7.4507 | -7.0555 | -5.9475 | -6.6913 | -8.7051 |
| 35 | -8.2969 | -6.9330 | -6.0582 | -7.5530 | -5.4655 | -7.2150 | -8.2814 | -4.0587 | -7.6792 | -7.9982 |
| 36 | -6.9151 | -7.8863 | -8.6444 | -7.2948 | -6.9246 | -9.1050 | -8.3755 | -7.2876 | -8.1041 | -7.0567 |
| 37 | -6.9913 | -8.3124 | -6.5473 | -6.3248 | -6.8853 | -7.6316 | -5.8904 | -6.8163 | -8.4647 | -7.4578 |
| 38 | -6.9794 | -7.2662 | -6.0356 | -5.8784 | -6.9056 | -8.3647 | -7.0901 | -4.7383 | -7.2043 | -7.4638 |
| 39 | -7.9863 | -7.0639 | -6.9615 | -7.5483 | -5.3714 | -6.2998 | -7.0734 | -5.9189 | -7.5995 | -6.1784 |
| 40 | -7.0579 | -8.4040 | -8.8313 | -8.2041 | -7.5221 | -7.0805 | -6.9080 | -7.8720 | -4.4121 | -4.5157 |
